# Health Perceptions and Adopted Lifestyle Behaviors During the COVID-19 Pandemic: Cross-National Survey

**DOI:** 10.2196/23630

**Published:** 2021-06-01

**Authors:** Nandi Krishnamurthy Manjunath, Vijaya Majumdar, Antonietta Rozzi, Wang Huiru, Avinash Mishra, Keishin Kimura, Raghuram Nagarathna, Hongasandra Ramarao Nagendra

**Affiliations:** 1 Swami Vivekananda Yoga Anusandhana Samsthana University Bengaluru India; 2 Sarva Yoga International Sarzana SP Italy; 3 Shanghai Jiao Tong University Shanghai China; 4 Vivekananda Yoga China Shanghai China; 5 Japan Yoga Therapy Society Yonago City Japan

**Keywords:** health behavior, self-report, cross-national survey, COVID-19, behavior, perception, lifestyle, nutrition, real-time

## Abstract

**Background:**

Social isolation measures are requisites to control viral spread during the COVID-19 pandemic. However, if these measures are implemented for a long period of time, they can result in adverse modification of people’s health perceptions and lifestyle behaviors.

**Objective:**

The aim of this cross-national survey was to address the lack of adequate real-time data on the public response to changes in lifestyle behavior during the crisis of the COVID-19 pandemic.

**Methods:**

A cross-national web-based survey was administered using Google Forms during the month of April 2020. The settings were China, Japan, Italy, and India. There were two primary outcomes: (1) response to the health scale, defined as perceived health status, a combined score of health-related survey items; and (2) adoption of healthy lifestyle choices, defined as the engagement of the respondent in any two of three healthy lifestyle choices (healthy eating habits, engagement in physical activity or exercise, and reduced substance use). Statistical associations were assessed with linear and logistic regression analyses.

**Results:**

We received 3371 responses; 1342 were from India (39.8%), 983 from China (29.2%), 669 from Italy (19.8%), and 377 (11.2%) from Japan. A differential countrywise response was observed toward perceived health status; the highest scores were obtained for Indian respondents (9.43, SD 2.43), and the lowest were obtained for Japanese respondents (6.81, SD 3.44). Similarly, countrywise differences in the magnitude of the influence of perceptions on health status were observed; perception of interpersonal relationships was most pronounced in the comparatively old Italian and Japanese respondents (β=.68 and .60, respectively), and the fear response was most pronounced in Chinese respondents (β=.71). Overall, 78.4% of the respondents adopted at least two healthy lifestyle choices amid the COVID-19 pandemic. Unlike health status, the influence of perception of interpersonal relationships on the adoption of lifestyle choices was not unanimous, and it was absent in the Italian respondents (odds ratio 1.93, 95% CI 0.65-5.79). The influence of perceived health status was a significant predictor of lifestyle change across all the countries, most prominently by approximately 6-fold in China and Italy.

**Conclusions:**

The overall consistent positive influence of increased interpersonal relationships on health perceptions and adopted lifestyle behaviors during the pandemic is the key real-time finding of the survey. Favorable behavioral changes should be bolstered through regular virtual interpersonal interactions, particularly in countries with an overall middle-aged or older population. Further, controlling the fear response of the public through counseling could also help improve health perceptions and lifestyle behavior. However, the observed human behavior needs to be viewed within the purview of cultural disparities, self-perceptions, demographic variances, and the influence of countrywise phase variations of the pandemic. The observations derived from a short lockdown period are preliminary, and real insight could only be obtained from a longer follow-up.

## Introduction

The World Health Organization (WHO) declared the outbreak of COVID-19 a pandemic on March 11, 2020 [1]. As of March 24, 2020, the most affected regions in the world were the Western Pacific region (China, the Republic of Korea, Japan, etc), with a total of 96,580 reported confirmed cases, and the European region (Italy, Spain, Germany, the United Kingdom, etc), which accounted for a total of 195,511 positive cases [2]. There was a global panic due to the shifting of the COVID-19 epicenters from China to Europe, mainly Italy, which reported the worst outcomes up to March 25, 2020 (69,176 reported cases and the maximum number of COVID-19 deaths of 6820) [2].

Global disease outbreaks impact varied aspects of physical and mental health, even suicidality [3-5]. As observed in the infectious disease epidemic of severe acute respiratory syndrome (SARS) in 2003, exposure to new pathogens can manifest as a qualitatively distinct mental impact [6]. Social isolation measures (large-scale quarantines, long-term home confinements, and nationwide lockdowns) [7-11], although essential for controlling viral spread, go against the inherent human instinct of social relationships [12,13]. If these measures are implemented for a long duration, they can be detrimental to mental health, as observed in recent reports from China and Vietnam [14-17], and they are expected to result in modification of people’s lifestyle behaviors, such as increased adoption of unhealthy dietary habits and sedentary behavior. These changes can exacerbate the burden of the “pandemics” of behavioral and cardiovascular diseases that already prevail in modern societies [18,19]. The latest trends of re-emergences of such infectious disease outbreaks merit timely preparedness involving community engagement and focus on healthy lifestyle behaviors [20,21]. Although the mental impact of the COVID-19 pandemic is being addressed in a timely fashion [22,23], the associated real-time influences on people’s health perceptions and lifestyle choices remain underresearched [24,25]. Careful consideration of the demographic and cultural impact of tailored public health intervention strategies on human behavior is also greatly needed when designing such strategies. Here, we report the findings of a cross-national survey that aimed to generate rapid perspectives on the status of health-related perceptions and their influence on the likelihood of adoption of healthy lifestyle choices during the COVID-19 pandemic. The settings were China and Japan, two nations in the Western Pacific region that were greatly impacted by COVID-19; Italy, from the European region; and India, a highly populous South Asian country that was a potential threat region at the time of the survey [2,7-9,11].

## Methods

### Sampling and Data Collection

Given the restricted mobility restrictions and confinement due to the COVID-19 lockdown, we conducted a cross-sectional survey using a web-based platform. We disseminated the survey through the circulation of a Google Form via institutional websites and private social media networks, such as Facebook and WhatsApp. We also used the group email lists of a few social organizations, universities, academic institutions, and their interconnections to share the questionnaire links, which further facilitated the snowball sampling. The respondents were residents of China, Japan, Italy, and India who were aged 18 years or older. We anonymized the data to preserve and protect confidentiality. The study was approved by the institutional review boards and institutional ethics committees of the respective nations: Swami Vivekananda Yoga Anusandhana Samsthana (SVYASA), India; Sarva Yoga International, Italy; Shanghai Jiao Tong University, China; and Japan Yoga Therapy Society, Japan. Respondents were informed about the objectives of the survey and the anonymity of their responses. Informed consent was obtained through a declaration of the participants of their voluntary participation, the confidentiality of the data, and the use of the collected information for research purposes only. The survey period was April 3-28, 2020. Once submitted, the responses were directly used for the analysis, and revisions of the responses were not allowed.

### Questionnaire Structure

We chose a short format for the questionnaire, with 19 questions to facilitate rapid administration. The first set of questions (Q1-Q5) were related to the respondents’ demographic details: age, gender, country of residence, working status, and the presence of any chronic illness or disability diagnosed by a physician. The next set (Q6-Q14) contained perception-related questions on self-rated physical and mental health, sleep quality, coping ability, energy status (a psychological state defined as an individual's potential to perform mental and physical activity [26,27]), coping flexibility, and perceptions related to interpersonal relationships as well as the fear of the pandemic. The questions were phrased as statements, with responses recorded on 3- or 5-point scales. For example, the respondents were requested to self-rate their mental and physical health status with the questions “How do you rate your physical health at present as” and “How do rate your mental health at present as” with answer modalities of (1) excellent, (2) very good, (3) good, (4) average, and (5) poor. These single-item self-health assessment questions are validated tools used in national surveys and epidemiological studies to assess health perceptions among individuals, strongly related to various morbidities, and mortality, and they have been validated across various ethnicities [28-33]. A further set of questions (Q15-Q19) focused on items related to the respondents’ recent lifestyle behavior choices: eating habits, engagement in physical activity or exercise, and substance use. Permitted responses for these behavior-related questions were either yes or no. For eating habits, the respondents provided self-rated scores for their time of eating; nourishment related to intake of vegetables and fibers; and daily intake of “junk food” (described as packaged and processed sweets or salty snacks); the combined scores were dichotomized into “good” (score ≥3) and “poor” (score ≤2).

### Data Analysis

An exploratory factor analysis using the principal axis factoring and varimax rotation suggested that three factors were present in the data. Items related to health perceptions were used to form a scale for perceived health status (the health scale); the scores were represented as mean (SD). For the remaining two factors, we could not form scales, as they scored Cronbach α values <.6; instead, we used the most relevant single item to represent the factor. The two primary outcomes of the study were the health scale and the adoption of healthy lifestyle choices. The health scale was derived as mentioned above; further health scale scores were categorized based on tertile distribution into low (poor), middle (average), and high (good) scores. Adoption of healthy lifestyle choices was defined as the engagement of the respondent in any two of three healthy lifestyle choices (eating habits, substance use, and exercise). Multivariate linear and logistic regression analyses were used to test the influence of the perceptions and the personal variables on the primary outcomes. Most of the items in the survey were recorded as 3-point responses. Hence, to achieve homogeneity in the analyses of the survey items, the 5-point Likert responses of the self-rated health items, excellent, very good, good, average, and poor, were collapsed into three categories: (1) very good/excellent, (2) good, and (3) average/poor. Analysis of variance was used to assess comparisons between continuous variables, and *P*<.05 was considered significant. Chi-square analysis was used for cross-country comparisons for categorical variables.

## Results

The aim of this survey was to understand the cross-national psychosocial and behavioral impact of the lockdowns and social isolations imposed due to the COVID-19 pandemic. We received 3370 responses: 1342 from India (39.8%), 983 from China (29.2%), 669 from Italy (19.8%), and 377 from Japan (11.2%). The demographic profiles of the respondents are presented in Table 1.

**Table 1:** Countrywise representation of the personal characteristics of the survey participants.

Variable		Overall (N=3371)	India (n=1342)	China (n=983)	Japan (n=377)	Italy (n=669)		*P* value^a^
Age (years), mean (SD)		36.04 (15.54)	29.42 (12.29)	29.77 (11.98)	53.49 (9.35)	48.43 (13.65)		<.001
**Age group (years), n (%)**								<.001
	18-24	1200 (35.6)	685 (51.0)	490 (49.8)	1 (0.3)		31 (4.7)	
	25-34	503 (14.9)	267 (19.9)	152 (15.5)	4 (1.1)		84 (12.5)	
	35-54	1176 (34.9)	330 (24.6)	314 (32.0)	217 (57.5)		309 (46.2)	
	55-64	330 (9.8)	40 (3.0)	21 (2.1)	98 (26.0)		169 (25.2)	
	>65	162 (4.8)	20 (1.5)	6 (0.6)	57 (15.1)		76 (11.4)	
Female gender, n (%)		2535 (75.2)	880 (65.6)	802 (81.6)	348 (92.0)		506 (75.6)	<.001
Working, n (%)		1709 (50.7)	582 (43.4)	406 (41.3)	335 (89.0)		395 (59.0)	<.001
Has a chronic illness, n (%)		647 (19.2)	169 (12.6)	84 (8.5)	151 (40.0)		314 (46.9)	<.001

^a^Cross-country comparisons for categorical variables were conducted using chi-square analysis. Analysis of variance was conducted to assess comparisons among the continuous variable of age. A *P* value <.05 was considered significant.

The mean age of the respondents was 36.04 years (SD 15.54) (Table 1); the average age of the Indian and Chinese respondents (29.42 years, SD 12.29, and 29.77 years, SD 11.98, respectively) was lower than that of the Japanese and Italian respondents (53.49 years, SD 9.35, and 48.43 years, SD 3.65, respectively). Overall, there was a higher representation of the female gender (2535/3371, 75.2%). Japan had the highest representation of women (348/377, 92.0%) and working people (335/377, 89.0%) (Table 1). Italy and Japan had the highest representations of respondents with a known status of chronic illness (314/669, 46.9%, and 151/377, 40.0%, respectively).

Table 2 shows the countrywise status of the perceptions of health and psychosocial factors reported in response to the ongoing outbreak of COVID-19. The health status score was highest for Indian respondents (9.43, SD 2.43) and lowest for Japanese respondents (6.81, SD 3.44). Overall, 846/3371 (25.1%) of the respondents had good health status; Japanese and Chinese respondents had the highest representation of low health status (236/377, 62.6%, and 562/983, 57.2%, respectively). Sleep quality was perceived well by the majority of Indians (917/1342, 68.3%), and the majority of Japanese and Chinese respondents perceived their sleep quality as average/poor (264/377, 70%, and 554/983, 56.3%, respectively). Italian respondents had almost equal representations of good and average sleep qualities. Coping abilities during social isolation were perceived as good by 1264/3371 (37.5%) of the overall population, with the countrywise trend of India (672/1342, 50.1%) > Italy (283/669, 42.3%) > Japan (131/377, 34.8%) > China (178/983, 18.1%). Fear response was almost equally distributed in positive or intermediate categories for most of the country respondents, except for Italians, among whom the intermediate or partial fear response was the most evident (469/669, 70.1%). Coping flexibility responses were very similar across all the countries except Japan, wherein the majority of respondents (317/377, 84.1%) reported experiencing little challenging response to sudden changes in living norms**.** Responses to interpersonal relationships followed the trend of India (733/1342, 54.6%) > Japan (183/377, 48.5%) > Italy (287/669, 42.9%) > China (337/983, 34.3%). Adopted lifestyle behavior yielded the trend of India (1129/1342, 83.9%) > Italy (361/669, 54.0%) > China (436/983, 44.4%) > Japan (137/377, 36.2%).

Based on the regression analysis on the perceived health status, female respondents had a 0.14 lower score compared to male respondents (Table 3). Participants with a positive history of chronic illness and those who were not working also had lower health status scores, by 0.11 and 0.04, respectively, compared to their counterparts. Increased personal relationships and positive fear response were associated with increases in health status across all the countries, particularly Japan, which showed the highest value of β (.60). For Indian respondents, an increase in age was significantly associated with increase in health status by a score of 0.12.

Increased interpersonal relationships was a significant predictor of adoption of health lifestyle choices across the respondents in all the countries except for Italy (adjusted OR 1.93, 95% CI 0.65-5.79) (Table 4). Positive perception of fear was significantly associated with likelihood of adoption of healthy lifestyle choices only in Indian respondents (adjusted OR 2.41, 95% CI 1.18-4.96). Perceived health status categories were significantly associated with the likelihood of adoption of healthy lifestyle choices across all the countries; most prominently, high health status increased adoption of healthy lifestyle choices by approximately 6-fold in China and Italy.

**Table 2:** Countrywise representation of perceptions and behavioral changes among the survey respondents related to the COVID-19 outbreak.

Perception or behavior and response			Overall (N=3371)		India (n=1342)		China (n=983)			Japan (n=377)		Italy (n=669)		*P* value^a^
**First factor^b^**														
	**Health status, mean (SD)**		8.26 (3.36)	9.43 (2.43)			7.09 ( 2.92)			6.81 (3.44)		8.43 (2.56)		.01
		High, n (%) Medium, n (%)	846 (25.1)	556 (41.4)			71 (7.2)			69 (18.3)		150 (22.4)		
			1062 (31.5)	413 (30.8)			350 (35.6)			72 (19.1)		225 (33.6)		
		Low, n (%)	1463 (43.4)	413 (30.8)			562 (57.2)			236 (62.6)		294 (43.9)		
	**Self-rated physical health, n (%)**													<.001
		Excellent/very good	1357 (40.2)		629 (46.9)		467 (47.5)			88 (23.3)		173 (25.9)		
		Good	1283 (38.1)		573 (42.7)		200 (20.3)			135 (35.8)		375 (56.0)		
		Poor/average	731 (21.7)		140 (10.4)		316 (32.1)			154 (40.8)		121 (18.1)		
	**Self-rated mental health, n (%)**													<.001
		Excellent/very good	944 (28.0)	645 (48.1)				0 (0)		93 (24.7)		206 (30.8)		
		Good	1670 (49.5)	535 (39.9)				642 (65.3)		122 (32.4)		371 (55.4)		
		Poor/average	757 (22.5)	162 (12.1)				341 (34.7)		162 (43.0)		92 (13.8)		
	**Self-rated sleep quality, n (%)**													<.001
		Good	1787 (53.0)			917 (68.3)			429 (43.6)		113 (29.9)		328 (49.0)	
		Average	1305 (38.7)			354 (26.4)			477 (48.5)		234 (62.1)		240 (35.9)	
		Poor	279 (8.3)			71 (5.3)			77 (7.8)		30 (8.0)		101 (15.1)	
	**Self-rated coping abilities, n (%)**													<.001
		Good	1264 (37.5)		672 (50.1)		178 (18.1)			131 (34.8)		283 (42.3)		
		Average	1492 (44.3)		539 (40.1)		516 (52.5)			139 (36.8)		298 (44.5)		
		Poor	615 (18.2)		131 (9.8)		289 (29.4)			107 (28.5)		88 (13.2)		
**Second factor** **, n (%)**														
	**Fear/anxiety related to COVID-19^c^**													<.001
		Not at all (positive)	1380 (40.9)		628 (46.8)		470 (47.8)			157 (41.6)		125 (18.7)		
		Partially (intermediate)	1829 (54.3)		662 (49.3)		485 (49.3)			213 (56.5)		469 (70.1)		
		Extremely (negative)	162 (4.8)		52 (3.9)		28 (2.8)			7 (1.9)		75 (11.2)		
	**Self-perception of low energy**													<.001
		Never	1449 (43.0)		667 (49.7)		282 (28.7)			239 (63.4)		261 (39.0)		
		Sometimes	1835 (54.5)		641 (47.8)		672 (68.4)			132 (35.0)		390 (58.3)		
		All the time	87 (2.6)		34 (2.5)		29 (3.0)			6 (1.6)		18 (2.7)		
	**Challenging response to sudden changes in living norms (coping flexibility)**													<.001
		Least/not at all/little	845 (25.1)	436 (32.5)			221 (22.5)			44 (11.7)		144 (21.5)		
		Little	1454 (43.1)	417 (31.1)			411 (41.8)			317 (84.1)		309 (46.2)		
		Extremely/somewhat	1072 (31.8)	489 (36.4)			351 (35.7)			16 (4.2)		216 (32.3)		
**Third factor, n (%)**														
	**Interpersonal relationships^c^**													<.001
		Increased	1540 (45.7)		733 (54.6)		337 (34.3)			183 (48.5)		287 (42.9)		
		Not changed	1572 (46.6)		533 (39.7)		550 (56.0)			179 (47.5)		310 (46.3)		
		Reduced	259 (7.7)		76 (5.7)		96 (9.8)			15 (4.0)		72 (10.8)		
	**Motivating influence of COVID-19 on lifestyle**													<.001
		Completely	1175 (34.8)		605 (45.1)		217 (22.1)			132 (35.0)		221 (33.0)		
		Partially	1919 (57.0)		641 (47.8)		695 (70.7)			223 (59.2)		360 (53.8)		
		Not at all	277 (8.2)		96 (7.1)		71 (7.2)			22 (5.8)		88 (13.2)		
	**Adoption of ≥2 healthy lifestyle choices**		2643 (78.4)		1126 (83.9)		750 (76.3)			283 (75.1)		485 (72.5)		<.001
		Adoption of healthy eating behavior	1801 (53.4)		867 (64.6)		436 (44.4)			137 (36.3)		361 (54.0)		<.001
		Decreased dependency on and use of tobacco, alcohol, or any other substances	3173 (94.1)		1277 (95.2)		918 (93.4)			355 (94.1)		623 (93.1)		<.001
		Increased engagement in exercise or similar activities	2280 (67.6)		910 (67.8)		672 (68.4)			272 (72.1)		426 (63.7)		<.001

^a^Cross-country comparisons for categorical variables were conducted using chi-square analysis; all the *P* values were significant.

^b^An exploratory factor analysis using principal axis factoring and varimax rotation suggested that there were 3 factors present in the data. The first factor consisted of health-related perceptions; composite scores for perceived health were generated as summative scores of the included items.

^c^For the remaining 2 factors, scales could not be formed; rather, the single items that were thought to best summarize the respective factors were considered for further association analyses.

**Table 3:** Multivariate linear regression analysis (β coefficients, standard errors, and *t* and *P* values) of the association between health status, personal variables, and perceptions.

Predictors			Overall				India				China				Japan				Italy			
			β	SE	*t*	*P*	β	SE	*t*	*P*	β	SE	*t*	*P*	β	SE	*t*	*P*	β	SE	*t*	*P*
**Demographic variables**																						
	Age		.14	0.01	5.12	<.001	.12	0.01	3.74	<.001	.07	0.01	1.79	.07	.08	0.02	1.55	0.12	–.07	0.02	–0.66	.51
	**Gender (reference: male)**																					
		Female	–.14	0.12	–7.51	<.001	–.09	0.14	–3.24	<.001	–.01	0.23	–0.35	.72	.01	0.64	–0.30	0.77	<.001	0.52	–0.03	.97
	**Working status (reference: working)**																					
		Not working	–.04	0.13	–2.04	.04	–.01	0.15	–0.32	.75	–.02	0.23	–0.54	.59	–.04	0.56	–0.71	0.48	–.03	0.55	–0.36	.72
	**Chronic illness (reference: no)**																					
		Yes	–.11	0.15	–5.63	<.001	–.16	0.20	–6.12	<.001	–.06	0.31	–2.04	.04	–.14	0.35	–2.81	0.01	–.09	0.47	–0.96	.34
**Perceptions**																						
	**Interpersonal relationships (reference: decreased)**																					
		Increased	.37	0.21	10.76	<.001	.38	0.28	6.48	<.001	.21	0.31	4.12	<.001	.60	0.85	4.86	<.001	.27	0.68	2.17	.03
		No change	.14	0.21	4.15	<.001	.21	0.29	3.71	<.001	.05	0.29	1.08	.28	.33	0.84	2.66	0.01	019	0.66	1.56	.12
	**Fear response (reference: poor)**																					
		Positive	.54	0.30	10.84	<.001	.59	0.33	8.69	<.001	.71	0.52	8.02	<.001	.54	1.38	2.72	0.01	.50	1.02	3.03	<.001
		Fair	.29	0.30	5.82	<.001	.35	0.33	5.22	<.001	.38	0.51	4.35	<.001	.26	1.37	1.30	0.20	.30	0.97	1.77	.08

**Table 4:** Role of perceptions in the adoption of healthy lifestyle choices.

Perception			Overall		India		China		Japan		Italy	
			OR^a^ (95% CI)	Adjusted^b^OR (95% CI)	OR (95% CI)	Adjusted OR (95% CI)	OR (95% CI)	Adjusted OR (95% CI)	OR (95% CI)	Adjusted OR (95% CI)	OR (95% CI)	Adjusted OR (95% CI)
**Health status (reference: low)**												
	High		3.67 (2.87-4.68)	3.42 (2.51-4.64)	2.98 (2.07-4.28)	2.62 (1.75-3.92)	6.02 (2.38-15.20)	5.83 (2.30-4.79)	3.64 (1.59-8.37)	2.83 (1.18-6.77)	3.33 (2.01-5.51)	6.22 (1.90- 20.40)
	Medium		2.09 (1.72-2.54)	2.00 (1.59-2.50)	1.76 (1.24-2.50)	1.57 (1.07-2.31)	2.61 (1.85-3.69)	2.43 (1.72-3.45)	1.33 (0.72-2.45)	1.06 (0.54-2.08)	2.10 (1.42-3.12)	2.46 (1.03-5.83)
**Interpersonal relationships^c^ (reference: decreased)**												
	Increased		2.21 (1.64-2.98)	2.42 (1.70-3.45)	1.86 (1.03-3.37)	2.16 (1.15-4.08)	2.01 (1.18-3.41)	1.77 (1.03-3.05)	4.43 (1.49-13.15)	5.25 (1.46-8.92)	1.86 (1.07-3.22)	1.93 (0.65-5.79)
	Not changed		1.25 (0.94-1.7)	1.18 (0.84-1.66)	1.09 (0.60-1.97)	1.18 (0.63-2.21)	1.03 (0.64-1.68)	0.99 (0.61-1.62)	1.87 (0.65-5.42)	1.88 (0.54-6.52)	1.59 (0.93-2.73)	1.40 (0.50-3.96)
**Fear response^c^ (reference: poor)**												
	Positive		2.43 (1.69-3.50)	2.50 (1.54-4.05)	2.72 (1.38-5.36)	2.41 (1.18-4.96)	2.38 (1.06-5.33)	2.18 (0.96-4.94)	1.84 (0.34-9.99)	4.85 (0.73-32.19)	1.62 (0.86-3.04)	2.20 (0.41-11.71)
	Fair		1.36 (0.95-1.93)	1.33 (0.83-2.14)	1.37 (0.71-2.65)	1.32 (0.65-2.65)	1.46 (0.66-3.23)	1.32 (0.59-2.96)	0.93 (0.18-4.93)	1.97 (0.31-12.55)	1.34 (0.80-2.27)	1.25 (0.27-5.80)

^a^OR: odds ratio.

^b^Adjusted for sex, age, work status, and history of chronic illness.

^c^Factor represented by a single item that was thought to best represent the underlying notion.

## Discussion

The aims of this short cross-national behavioral survey study were to generate rapid ideas regarding perspectives on health and lifestyle behavior and to provide initial insights into designing global but culturally tailored public health policies.

### Health Perceptions: Countrywise Status

A differential countrywise response was observed toward perceived health status across the survey participants; Indians had a better representation of high health status (41.4%) compared to respondents from other countries (China, 7.2%, Japan, 18.2%, and Italy, 22.5%). Despite the inconsistencies in health perceptions, there was a consistent influence of social support measured by perceptions of interpersonal relationships and fear of perceived health status. However, there were countrywise differences in the magnitude of the impact of perceptions on health status; perception of interpersonal relationships was most pronounced in the comparatively older Italian and Japanese respondents (β=.68 and .60, respectively) and that of fear in the Chinese respondents (β=.71). These findings favor the implementation of regularized virtual interpersonal interactions toward combating the adverse health impact of the pandemic, particularly in countries with a higher proportion of older people [34]. Controlling the fear response through counseling would also aid the improvement of health outcomes in populations affected by pandemics. The findings of this survey related to the influence of gender on health perceptions (the health status score of female respondents was lower by 0.14 units compared to that of male respondents) are in line with the global trend of poorer health perception in women than in their male counterparts [35]. These real-time findings observed during the pandemic also relate with reports documented before the COVID-19 pandemic, with a generally higher prevalence of adverse mental health symptoms in women compared to men [36]. Overall, there seemed to be a differential influence of demographic variables on health perceptions across the global population during the pandemic.

The comparatively high scores of the perceived health status in Indian respondents could be underlined by an early phase of the pandemic with slower progression in India during the survey period [11]. The younger age of the Indian respondents (mean age 29.42 years, SD 12.29) seemed to further facilitate interpersonal relationships (54.6%) during the lockdown, which also explains their better health status (β=.38) [34,37]. Younger age identity has been associated with well-being and better perceptions of health [38]. However, in this survey, an unexpectedly positive linear relationship was observed between increasing age and better perception of health status (β=.12) in young Indian respondents. This finding can be attributed to the compounding effect of the COVID-19 pandemic on already existing emotional distress among young adults (related to their examinations, uncertainties, social relationships, etc) [39].

Unfortunately, in line with previous reports [14,15], we could also observe a continued/posttraumatic impact of the pandemic in Chinese respondents, reflected in their comparatively low perception of health status (poor health status was reported by 57.2% of these respondents). We believe the poor health perceptions in the Chinese respondents is due to the underlying influence of fear perceptions (β=.71). Further, since the country had successfully emerged from the first wave of the pandemic during the survey, and social norms had also almost returned to normal, with fewer imposed lockdowns, the moderate increase in interpersonal relationships (34.3%) may not be sufficient to facilitate health status.

The observed low status of perceived health in the Japanese respondents (low health status, 62.6%) is in accord with a health paradox in that country, which is a tendency to perceive health poorly despite the advanced economy [40,41]. Although this influence is not direct, an indirect influence of the comparatively old, middle-aged demographic profile of the Japanese respondents along with the mediatory impact of chronic diseases on health status (β=–.14) could also underlie the lower health perceptions of the Japanese respondents [42]. The perception of poor sleep quality in the Japanese respondents also needs attention, as this finding is in line with reports of the suicidal tendencies in this country [43].

On a positive note, amid the aggravated pandemic at the time of the survey, the majority of the Italian respondents who were middle-aged perceived only partial fear of the pandemic (70.1% response), and they reported better health perceptions (health status score 8.43, SD 2.56) than Japanese respondents (health status score 6.81, SD 3.44) and Chinese respondents (health status score 7.09, SD 2.92). Approximately 55% of the responses for self-rated physical and mental health were in the moderate/fair tier, which is in accord with the reported tendency of Italian people toward intermediate categories of health perception [44]. The lack of negative influence of middle age and chronic illness on health perception can be attributed to the highly efficient medical care and adequate access to social support provided in Italy during the lockdown (improved interpersonal relationships were reported by 42.9% of Italian respondents).

### Role of Perceptions in the Adoption of Lifestyle Choices: Countrywise Comparisons

Despite the imposed social isolation and home confinement and the prevailing fear during the COVID-19 pandemic, we observed a positive behavioral response toward lifestyle. Overall, 78.4% of the respondents adopted at least 2 healthy lifestyle choices during the COVID-19 pandemic. The majority of the respondents (67.6%) reported increased engagement in physical activity or exercise as opposed to the expected sedentary behavior due to home confinement. This favorable although unexpected outcome can be attributed to the timely release of the advisory recommendations made by various global and government agencies, including the WHO, on home-based or other easy‐to‐perform exercises under physical restrictions [45,46]. One of the crucial affirmative responses observed in this survey was the overwhelming response toward substance use (94.1%), which is more justifiable by lack of availability [47] than motivational influence. Along similar lines, in a recent survey on the immediate response to COVID-19, a 3% reduction in smoking was reported in Italians, which was attributed to the fear of increased risk of respiratory distress or mortality [48]. To this end, we suggest the implementation of internet-based and cost-effective behavioral therapies, particularly cognitive behavioral therapy, which may aid the successful alleviation of maladaptive coping tendencies, thereby reducing the risk of future health catastrophes in the post–COVID-19 era [49,50].

Social connectedness is an important dimension that controls population health and healthy lifestyle behavior [51]. In this cross-national survey, perception of increased social support and capital, manifested through enhanced interactions among close friends and family members (measured as interpersonal relationships in the survey), seemed to fill the void of missing social connectedness and encouraged the adoption of healthy lifestyle choices (adjusted OR 2.42, 95% CI 1.70-3.45). The substantial representation of the adoption of healthy lifestyle choices in Chinese and Japanese respondents (~75%), irrespective of their overall poor health perceptions, could be related to reverse causality. In the Japanese respondents (who had an older, middle-aged demographic profile), their working status (OR 4.37, 95% CI 1.19-16.02) (Table S1, Multimedia Appendix 1) and interpersonal relationships (OR for the adoption of healthy lifestyle choices 5.25, 95% CI 1.46-18.92) also seemed to contribute significantly to the adoption of healthy lifestyle behavior.

The influence of interpersonal relationships on the adoption of healthy lifestyle choices was not consistent across different countries and was absent in the Italian respondents. However, this finding aligns with the previously reported relationship between a healthy lifestyle and self-perceived health in the European population [52]. Perception of good health was a prominent predictor of adoption of a healthy lifestyle (adjusted OR 6.22, 95% CI 1.90-20.40) in the middle-aged Italian respondents, with a 36.6% proportion of older individuals (>55 years). Even intermediate scores of health perceptions (health status) also significantly predicted the likelihood of the adoption of healthy lifestyle choices (OR 2.43, 95% CI 1.72-3.45) in the Chinese respondents compared to the respondents from other countries, explained by their demographic characteristic of younger age. These countrywise differential cultural influences of perceptions on health and health behaviors during pandemics indicate that endorsement of the same, such as family support and togetherness, should consider existing disparities, especially for western countries [13].

The findings of this report, particularly those regarding varied health perceptions and their differential influence on the likelihood of adopting healthy lifestyle choices, should be considered within the purview of the survey period with countrywise phase variations of the pandemic. Chinese respondents displayed the continued impact of the pandemic, as they had already witnessed one phase of the pandemic [2]. Younger Indian respondents scored better for their health- and behavior-related perceptions due to the stable and early phase of the pandemic (as of April 22, there was a comparatively steady expansion of COVID-19 cases in India compared to other countries, with 18,985 confirmed cases [11]). However, the responses of Japanese and Italian respondents related to their older age; these countries were also witnessing rising waves of COVID-19 at the time of the survey [7,53]. Japan was under an extended state of national emergency, as the number of “untraceable” cases was soaring [7]. Italy was also under an extended period of lockdown and was one of the hardest-hit nations, with an apparent mortality rate of approximately 13% [53,54].

The observed predominantly female participation in the survey indicates a lack of stringent sampling but also highlights the active involvement of women, who are considered to be at high risk of socioeconomic vulnerability toward disease outbreaks such as the COVID-19 pandemic. The positive response for self-care in women is also a sign of improving gender equity toward health awareness. The observed overwhelmingly female participation level (75.2%) could not be ascribed to the gender representation of countries such as India and China [55] but could be ascribed to the high readiness of the female population to interactively use the internet, in particular to research health-related information and programs, as observed in recent reports [56-58].

The study is limited by the lack of inclusion of perceptions of preventive behaviors and did not compare the respondents’ views on precautionary measures, such as the use of face masks [59]. In a recent cross-country comparison between Polish and Chinese respondents, higher use of face masks in Chinese respondents (Polish respondents, 35.0%; Chinese respondents, 96.8%; *P*<.001) was found to be associated with better physical and mental impact of the COVID-19 pandemic [59]. Further, the observations of the adopted lifestyle choices presented here are derived from a short lockdown period during the COVID-19 pandemic and are preliminary, influenced mostly by self-perception; demographic and cultural differences and realistic insight could only be obtained from a longer follow-up. Due to the self-reported nature of the observations, positive behavioral responses toward lifestyle are likely to be inflated.

Good perceived health was associated with improved interpersonal relationships. Older respondents were least likely to report a positive relationship change, as observed in the responses of Italian and Japanese survey participants. However, there was a strong influence of improved interpersonal relationships on perceived health as well as adoption of healthy lifestyle choices in Japanese respondents. These findings indicate the potential of regularized virtual interpersonal interactions to attenuate the adverse psychosocial impact of such pandemics.

In conclusion, the key finding of the survey is that the consistent positive influence of increased interpersonal relationships and good perceptions of health were found to have a significant influence on adopted lifestyle behaviors during the adverse time course of the COVID-19 pandemic. These favorable behavioral perceptions should be bolstered through enhanced health awareness, and regularized virtual interpersonal interactions, particularly in countries with an overall middle-aged or older population. Simultaneously, controlling the fear response through counseling would also help improve health outcomes in nations affected by pandemics. However, the observed human behavior has cultural influences, and it may not be globally generalizable.

### Data Availability Statement

The data that support the findings of this study are available on request from the corresponding author.

## Appendix

Multimedia Appendix 1 Supplementary table.

## Abbreviations

SARS: severe acute respiratory syndrome
SVYASA: Swami Vivekananda Yoga Anusandhana Samsthana
WHO: World Health Organization
