# Supplementary material for: Health Perceptions and Adopted Lifestyle Behaviors During the COVID-19 Pandemic: Cross-National Survey
Source: JMIR Form Res. 2021 Jun 1;5(6):e23630. doi: 10.2196/23630 (PMC8171386; doi:10.2196/23630)
Supplement: Multimedia Appendix 1 [file formative_v5i6e23630_app1.docx]

**Health Perceptions and Adopted Lifestyle Behaviors During the**

**COVID-19 Pandemic: Cross-National Survey**

Manjunath NK, Ph.D., Vijaya Majumdar^*^, Ph.D,, Nagarathna R, MD, Wang Huiru, Ph.D., Nagendra HR, Ph.D.

**Supplementary material**

**Appendix**

**Perception scales used in the analyses, with examples of items**

1. **Health indicators (4items)**

How do you rate your physical health at present as (How do you rate your mental health at present as?

How do you rate your sleep quality? Cronbach’s α 0.81)

1. **Anxiety/fear** (3 items)

Scale could not be formed, since cronbach’s α score was less than 0.6, the most relevant single item was used

***Is there a fear that worries you now?***

How challenging is it to accept a sudden change in the norms to live

Do you feel you are low in energy and down-hearted at present?

1. **Interpersonal relationships**

Do you agree that the social isolation has helped you to explore yourself and spend time with dear ones?

**Table S1. Role of subjective health indicators on lifestyle behavior**

|  |  |  | **Overall** |  | **India** | | | **China** | | | **Japan** | | | **Italy** | |  | | |
| --- | --- | --- | --- | --- | --- | --- | --- | --- | --- | --- | --- | --- | --- | --- | --- | --- | --- | --- |
|  |  | OR | 95% CI | P value | OR | 95% CI | P value | OR | 95% CI | P value | OR | 95% CI | P value | OR | 95% CI | | P value |  |
|  | **Age** | 1.00 | 0.99-1.01 | 0.74 | 1.03 | 1.02-1.04 | 0.06 | 1.01 | 1.01-1.03 | 0.11 | 1.00 | 0.97-1.04 | 0.77 | 0.98 | 0.95-1.02 | | 0.52 |  |
|  | **Gender** |  |  |  |  |  |  |  |  |  |  |  |  |  |  | |  |  |
|  | Female | 1.18 | 0.93-1.49 | 0.16 | 1.27 | 0.88-1.83 | 0.20 | 1.24 | 0.83-1.85 | 0.29 | 1.22 | 0.47-3.23 | 0.68 | 1.80 | 0.74-4.34 | | 0.20 |  |
|  | Male | Ref |  |  | Ref |  |  | ref |  |  | ref |  |  | Ref |  | |  |  |
|  | **Working status** |  |  |  |  |  |  |  |  |  |  |  |  |  |  | |  |  |
|  | Yes | 1.03 | 0.80-1.27 | 0.96 | 1.09 | 0.72-1.63 | 0.69 | 1.01 | 0.67-1.58 | 0.88 | 4.37 | 1.19-16.02 | 0.03 | 2.26 | 0.95-7.27 | | 0.11 |  |
|  | No | Ref |  |  | Ref |  |  | Ref |  |  | ref |  |  | Ref |  | |  |  |
|  | **Chronic illness** |  |  |  |  |  |  |  |  |  |  |  |  |  |  | |  |  |
|  | **Yes** | 0.99 | 0.74-1.30 |  | 0.74 | 0.45-1.19 | 0.21 | 1.21 | 0.68-2.17 | 0.51 | 0.54 | 0.29-0.99 | 0.05 | 1.29 | 0.55-3.03 | | 0.56 |  |
|  | No | Ref |  |  | Ref |  |  | Ref |  |  | ref |  |  | Ref |  | |  |  |
